# An Epidemic of Tetanus

**Published:** 1883-12

**Authors:** Alfred Sheen

**Affiliations:** Surgeon Glamorgan and Monmouthshire Infirmary, Cardiff


### AN EPIDEMIC OF TETANUS.

BY

ALFRED SHEEN, M.D.,

*Surgeon Glamorgan and Monmouthshire Infirmary, Cardiff.*

---

I THINK there can be no doubt that tetanus occurs, from time to time, in what may be called an *epidemic* form; and, if this be so, our enquiries regarding this distressing disease may be directed into such channels as shall ultimately result in a more satisfactory knowledge of its etiology, pathology, and treatment.

There has recently been at the Cardiff Infirmary a succession of cases of traumatic tetanus, and such an experience, although perhaps not new, is sufficiently rare to attract one's attention and to be interesting to the profession.

On the evening of May 21st, 1883, a labouring man, age 25, was admitted under the care of my colleague, Dr. Vachell, with acute and severe traumatic tetanus, which had come on suddenly with "lock-jaw" in the afternoon. His injury was a "smash" of two fingers and thumb, sustained on the 16th, and he was treated as an out-patient till his admission. On the morning of the 22nd the injured parts were amputated; the tetanus, continuing, was treated by chloral and bromide of potassium, and the patient died at 5 a.m. on the 23rd.

On May 23rd I had four major amputations—two legs below the knee in the same patient, for a tram-car smash, one leg below the knee for epithelioma of the foot, and one arm below the shoulder, for a railway smash. On the 24th I performed ovariotomy on a patient who had been an inmate since the 8th. These five operations were on four patients, three of whom subsequently had tetanus, and two of these cases ended fatally.

I will call the case already alluded to as being admitted with tetanus, "Case 1."

*Case* 2 was a labourer, single, age 18, admitted May 23rd at 5 a.m., with compound fractures of the right forearm and arm and severe crushing of soft parts, caused by both wheels of an engine passing over his arm. Primary amputation was performed below the shoulder-joint. On the 27th he was up and out in the garden for a short time, it being a beautifully fine day. On the 28th he was up but not out. On the 29th he complained of some difficulty in opening his mouth and in swallowing, severe acute tetanus supervened, and he died at 8 a.m. on the morning of the 31st. His morning and evening temperature up to the morning of the 30th, which was 24 hours after the first tetanic symptoms came on, was from 99° F. to 100° F., on the evening of the 30th it went up to 103° F., on the morning of the 31st it was 102° F., and ten minutes after death the thermometer recorded 104°.6 F. in the axilla.

*Treatment.*—Tinct. cannabis indicæ, ʒ ss. every hour.

*Case* 3, a labourer, age 40, single, was admitted on the same day as the last patient, suffering from severe compound fracture of both legs, caused by a tram-car going over them. Primary amputation of both legs, below the knee, was performed. He did well until June 3rd, when spasms came on in his lower extremities, and trismus was first noted on the 5th. He died from exhaustion on the 15th, being the thirteenth day of tetanus, the tetanic symptoms having somewhat subsided. His morning and evening temperature varied from 99° F. to 100° F. until the evening before his death (the 14th), when it was 104° F. On the morning of the 15th the pulse was 144 and the temperature 101° F.

(The other patient operated upon on the 23rd, and who did *not* suffer from tetanus, was treated in the general male ward, and was discharged cured three weeks after amputation).

*Case* 4 was an ovariotomy (in a patient aged 39) performed in a special large ward outside the Infirmary building, on May 24th; she was convalescent on June 1st, and was removed into the general female ward on June 7th. She complained slightly of her jaw on the 6th, and was moved back to her old quarters on the 11th. This was a sub-acute case, lasting about 22 days, but her life was often in jeopardy, and she was much troubled by profuse bronchitic secretion and cough, which latter was the main existing cause of her spasms. Her temperature remained normal for ten days after the onset of tetanic symptoms; during the next seven days it stood at about 100° F., going up to 103° F. on the 20th of June. After June 24th it became normal, and remained so.

Case 1 was placed in a medical ward and died in less than 36 hours from the commencement of tetanic symptoms. Cases 2 and 3 were both in the accident ward, and were both admitted and operated upon on May 23rd. Case 2 was seized with tetanic symptoms on May 29th, two days after he had been up and out in the garden. He was much alarmed, because he knew that he had lock-jaw, and he was aware of the other patient dying from it. He was removed to a private ward for quiet, but died within 48 hours of the first onset of symptoms. It can scarcely be that *fear* had any influence in inducing the disease, because he was not frightened until *after* the lock-jaw had shown itself. Had his going out into the open air, with a temperature of 99°—100°, any causative influence? Some may think it was a little rash to let a man go into the open air so soon after an amputation near the shoulder joint. Was it? The weather was beautifully fine, and the man importunate. Some experienced authorities are of opinion that cases of amputation do better in fine warm weather, when dealt with in tents with the air blowing in from all directions. Nevertheless, it is not improbable that a *chill* may have been the immediate exciting cause of his tetanus, and I think the history of this case would lead me to hesitate in future about allowing a patient to go out *quite* so early after an amputation. Case 3 was a man of reserved temperament, and evidently not quite "all there." He gave one the impression, from the time of his admission, of a man who felt that he was doomed to die. The tetanic symptoms commenced on June 3rd. This was a case of sub-acute tetanus, in which one had a fair hope of recovery, but although the patient took plenty of liquid nourishment he died on June 15th, the thirteenth day of the disease, his tetanic symptoms having considerably abated in severity. It seems inexplicable almost why he should have died from exhaustion.

Excluding the first case admitted with tetanus, the onset of the tetanic symptoms dated respectively May 29th, June 3rd and June 6th.

The other casualties admitted into the Infirmary about this time were:—

May 22. M.; stab in side; in hospital two days.

May 22. M., æt. 37; ununited fracture of tibia; operation.

May 22. M., æt. 24; contusion of back.

May 22. M., æt. 48; epithelioma of foot; amputation.

May 25. F., æt. 5; fracture of femur.

May 27. M., æt. 22; rupture of radial artery and suppuration.

May 28. M., æt. 38; fracture of tibia and fibula.

May 29. M., æt. 29; destruction of eye.

May 29. F., æt. 30; fracture of femur.

June 1. M., æt. 50; compound fracture of femur; died in a few hours.

June 2. M., æt. 27; compound fracture of fingers, &c.

June 5. F., æt. 43; removal of fatty tumour in neck.

June 6. M., æt. 16; disease of ankle joint; amputation.

All the operations which I have referred to in this paper were dealt with under strict antiseptic precautions, and ether was the anæsthetic employed.

Tetanus is truly a mysterious disease. Our knowledge of its pathology and etiology is meagre enough, and, therefore, our treatment must be at all times uncertain, and its results in many cases unsatisfactory. Although it has been said to be more common after injuries to the thumb and fingers, it follows all kinds of accidents, trivial and severe; its accession seems to be uninfluenced by the season of the year, and as to the pathological changes, such as "intense congestion of the spinal cord and structureless exudations, especially in the grey matter,"* it is impossible to say how far they are the causes or the results of the disease.

Poland, in Holmes's *System of Surgery*, gives a table of 3,668 operations at Guy's Hospital in seven years, of which 23 were followed by tetanus; out of 594 wounds of all kinds there were 9 cases of tetanus, and out of 398 compound fractures there were also 9 cases.

* C. Macnamara: Quain's *Dictionary*, Art. Tetanus.

Macnamara* says "it seems certain that local circumstances and meteorological conditions greatly influence the occurrence of tetanus. It is seldom absent in the tropics. It is a common remark in Bengal that on sudden changes of temperature cases appear amongst the surgical patients." I scarcely know whether the cases I have mentioned will in any way bear out this statement. This I may say, as to supposed meteorological influences, that on May 22nd, the day before most of these cases were operated upon, the temperature was 71°.8, the previous day's temperature being 60°.6, and that of May 23rd 66°.2. Here was a sudden accession of heat, which I remembered afterwards, but it may be said that it did not occur at or about the time when tetanic symptoms showed themselves. I do not know whether it is so, but it is possible that tetanus may have a period of incubation, as it is said to run a definite course. The *barometer* at or about this period averaged 30°, and as to *direction of wind* it was most varied. There was no rain. I do not think any importance is to be attached to these various points, except, perhaps, the sudden rise of temperature.

The "local circumstances" at our Infirmary are these: the applications for admission are so numerous that a bed is scarcely allowed to get cool before another occupant has possession of it, and whenever the mysterious "epidemic influence" is in the ascendant, erysipelas is not unlikely to make its appearance in the wards and some of our nurses or resident officers suffer from general malaise, sore throat, boils, &c.

There are some points bearing on the *prognosis* of this disease which it is desirable to bear in mind.

1. Acute and severe cases are almost always fatal under any treatment.

* Art. cit.

2. "The more sudden the onset the more surely fatal is the disease. If it occur 22 days after the receipt of injury it is generally recovered from."*

3. The sub-acute cases, under careful general treatment and very careful nursing, may fairly be expected to recover.

4. It is stated that if the patients survive till the twelfth day they, as a rule, recover, and they may usually be pronounced cured in 25 days from the beginning (Macnamara); yet Poland† states that out of 327 cases 43 were fatal after a duration of from 10 to 22 days, 11 after 22 days, and that the longest duration on record of a fatal case was 39 days.

5. Macnamara‡ lays stress upon the temperature as a guide to the danger, or otherwise, of the patient. He says so long as the temperature is under 101° F. there is nothing to fear, if it rises above 101° F. there is imminent danger, and if it is 103° there is cause for the greatest anxiety. (Dr. O'Beirne records 200 cases without any fever at all.)

6. Those cases in which tetanus occurs in wounds already granulating seem to run a milder and more chronic course.

What is the *origin* of tetanus? The symptoms "are undoubtedly referable to an abnormal influence of the nervous centres which control the action of the voluntary muscles."§ True. But what are the conditions, or the causes, or the influences which bring about these symptoms in cases of surgical injury? Billroth|| places tetanus amongst the "group of diseases which belong to the traumatic and phlogistic states of infection." He "inclines

* Poland, loc. cit. † *Ibid*, loc. cit. ‡ Art. cit.

§ Macnamara, art. cit. || *Surgery*, vol. ii., p. 67, New Syd. Soc.

very much at present to the humoral theory of tetanus as a peculiar poison disease, without, however, being able to furnish proofs thereof." This is a peculiarly attractive theory, and it seems to me that it is in this direction that we might fairly look to physiological investigations to help us to some better knowledge of the nature of this terrible disorder rather than by investigations as to the state of the spinal nervous system after death, to which system all enquiries hitherto, in search of an answer to the question I have put, seem mainly to have been directed. We have the poison of rabies, producing, after an uncertain period of incubation, hydrophobia; we have the more tangible poison, strychnine, producing, in poisonous doses, symptoms somewhat resembling those of tetanus. May there not be also a poison of tetanus which, as Billroth puts it, "*perhaps very rarely and under entirely peculiar circumstances* becomes formed in the wounds and absorbed from thence?"

Dr. Ogston is in favour of the theory that tetanus is really a septic disease. His views are to be found in some able papers on micrococcus poisoning in the *Journal of Anatomy and Physiology*, vol. xvi., pts. iv. and v., and vol. xvii., pt. i. Unfortunately I have not those papers by me for reference.

As to *treatment*. In acute and severe cases, supervening quickly on the injury, the result is usually fatal whatever system of treatment is adopted. The late Dr. Anstie gave it as his opinion that "Curare,* Calabar Bean, and Nicotine, subcutaneously injected, seem the

* *Curare* impairs and destroys the conductivity of the motor nerves. *Tobacco* is a feeble paralyser of the motor nerves, but more decided paralyser of voluntary muscular fibre. *Calabar Bean* has the advantage, over these and other substances, of directly and powerfully diminishing the reflex activity of the cord. (Fraser: *Practitioner*, vol. i., p. 77.)

three really hopeful means of combating tetanus," and whether we adopt the poison theory or not, such treatment seems to be based on the sound principle of modifying and subduing the *effects* of the disease, and so prolonging the life of the patient with the hope that the disease will gradually lose its intensity and so finally pass away. Dr. Eben. Watson, of Glasgow, speaks hopefully of Calabar Bean. He gives a table of 18 cases so treated, with 10 recoveries and 8 deaths.* I am unable to ascertain how far these were acute and severe cases, except as to 6 of his own. Five of these were of this nature and all died, whilst the sixth, which recovered, had been suffering from tetanus for five days previous to admission to hospital and was not acute in its nature. Dr. Watson † has recorded a case of acute traumatic tetanus, with recovery, in which 1,026 grains of the alcoholic extract of Calabar Bean were given in all. He urges the safety of a very full and free administration, if only two conditions be fulfilled, viz.:—(1) careful and intelligent watching in giving it as often and in such a dose as may be required, and (2) supporting the strength well by fluid nourishment and stimulants. It should be commenced with as early as possible in the case, and the dose boldly increased till some decided effect is produced. ("Muscle itself produces, during its contraction, substances which cause it to contract.") ‡

As to the use of tobacco, Dr. Anstie says, "there are very many records scattered here and there which tend to prove that tobacco exerts a powerful influence in tetanus; but it is a most dangerous and unmanageable drug in any form except that of the subcutaneous injec-

* *Practitioner*, vol. iii., p. 158. † *Ibid*, vol. iv., p. 208.

‡ Fraser: *Practitioner*, vol. i., p. 83.

tion of nicotine; and I should much like to see this plan extensively tried. It has already produced brilliant results in more than one recorded case."*

In acute tetanus, then, in spite of many failures, the records of success are sufficiently numerous to induce us persistently to persevere in the full and frequent use of one of these remedies. Sub-acute and chronic cases are far more hopeful as to treatment. Here, I think, the indications are, clearly, to try and keep the patient alive from day to day by abundant fluid nourishment, and to induce sufficient sleep by the use of chloral alone or combined with bromide of potassium, and to moderate spasm by the use of these remedies and the occasional resort to chloroform and hypodermic injection of morphia. Further, I know of no disease where careful and trained nursing is more important as an aid to recovery; so many apparently trivial accidents may happen in the progress of the case in which *intelligent trained* nursing may possibly, nay probably, obviate death. Mr. Bryant† lays stress on the fact that "*as long as the slightest evidence of disease exists, a sudden spasm of the glottis may at any time destroy life.*" It is important to bear this in mind. I attribute the recovery of my case of ovariotomy in a large measure to the careful and unremitting attention bestowed upon it by our House-surgeon, Mr. P. Rhys Griffiths, and the two nurses who took charge of her day and night—the one who was in charge during the night being a thoroughly trained and skilful nurse.

In addition to the four cases already referred to in this paper, and subsequent to them, there have been four more in the wards of the Cardiff Infirmary to which I may briefly refer.

* *Practitioner*, vol. iii., p. 120.

† *Surgery*, vol. i., p. 242.

A woman, age 30, of drunken habits, was admitted on June 27th, under the care of my colleague, Dr. Vachell. She had sustained an accident to her left arm on May 31st, and decided symptoms of tetanus set in on June 24th, although she stated she felt some slight stiffness of the jaws on June 12th. This was evidently a sub-acute case, setting in some time after the receipt of injury, and a favourable result was prognosticated. She was treated by frequent hypodermic injections of morphia, with constant nourishment, and was discharged cured on July 20th.

A boy, age 14, admitted under my care on July 23rd, with smashed foot. Syme's amputation was performed at once, antiseptically. Tetanic symptoms set in on the morning of the 28th, and he died on the evening of the 29th.

A man, age 63, admitted under Dr. Vachell's care on August 23rd, with compound fracture of the left leg, which was dressed antiseptically. The fracture did well. Tetanic symptoms set in on September 26th and he died on September 28th, at 10 p.m.

A man, age 43, admitted under Dr. Taylor's care, during my temporary absence from home, on September 20th, with compound fracture of the leg. Dr. Taylor performed primary amputation below the knee. Tetanic symptoms set in on the 26th and he died at 9 p.m. on the 28th.

